# A Multi-Model Pipeline for Translational Intracerebral Haemorrhage Research

**DOI:** 10.1007/s12975-020-00830-z

**Published:** 2020-07-07

**Authors:** Sarah E. Withers, Adrian R. Parry-Jones, Stuart M. Allan, Paul R. Kasher

**Affiliations:** 1 Division of Neuroscience and Experimental Psychology, School of Biological Sciences, Faculty of Biology, Medicine and Health, Manchester Academic Health Science Centre, The University of Manchester, Oxford Road, Manchester, M13 9PT UK; 2 Division of Cardiovascular Sciences, School of Medical Sciences, Faculty of Biology, Medicine and Health, Manchester Academic Health Science Centre, The University of Manchester, Oxford Road, Manchester, M13 9PT UK; 3 Manchester Centre for Clinical Neurosciences, Salford Royal NHS Foundation Trust, Manchester Academic Health Science Centre, Stott Lane, Salford, M6 8HD UK

**Keywords:** Pre-clinical, Intracerebral haemorrhage, Disease models, Drug discovery

## Abstract

Apart from acute and chronic blood pressure lowering, we have no specific medications to prevent intracerebral haemorrhage (ICH) or improve outcomes once bleeding has occurred. One reason for this may be related to particular limitations associated with the current pre-clinical models of ICH, leading to a failure to translate into the clinic. It would seem that a breakdown in the ‘drug development pipeline’ currently exists for translational ICH research which needs to be urgently addressed. Here, we review the most commonly used pre-clinical models of ICH and discuss their advantages and disadvantages in the context of translational studies. We propose that to increase our chances of successfully identifying new therapeutics for ICH, a bi-directional, 2- or 3-pronged approach using more than one model species/system could be useful for confirming key pre-clinical observations. Furthermore, we highlight that post-mortem/ex-vivo ICH patient material is a precious and underused resource which could play an essential role in the verification of experimental results prior to consideration for further clinical investigation. Embracing multidisciplinary collaboration between pre-clinical and clinical ICH research groups will be essential to ensure the success of this type of approach in the future.

## Introduction

**Fig. 1:**
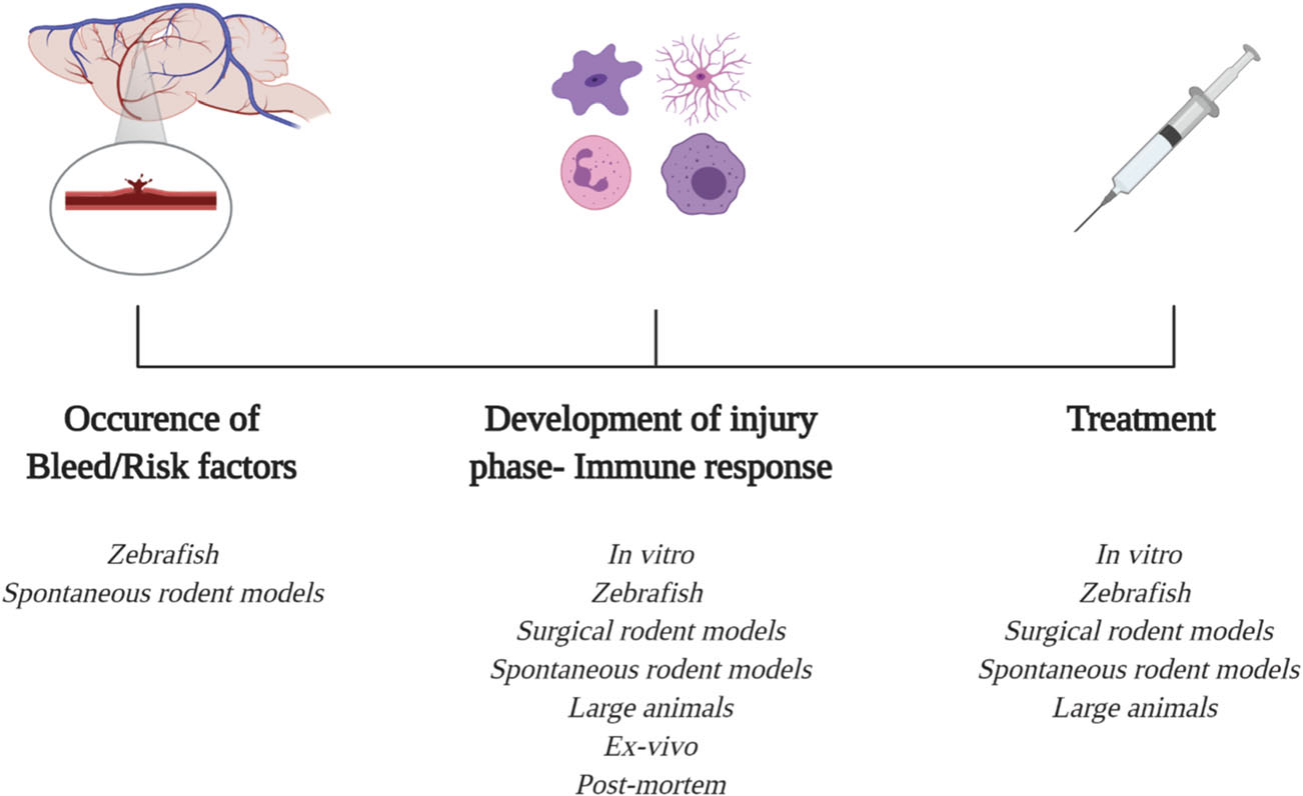
ICH timeline. This timeline represents the major factors which can be studied in pre-clinical ICH models/systems. Mechanisms that underpin ICH risk can be explored in spontaneous ICH models, such as hypertensive rodents and zebrafish models. Secondary injury progression can be readily observed in all the pre-clinical models, and observations can also be verified in patient tissue such as serum and post-mortem brains. Lastly, potential treatments can be tested across all pre-clinical animal and cell models, where novel candidate therapeutics could be identified through high-throughput screens (e.g. in vitro, zebrafish) and subsequently verified in a mammalian system

Stroke is the second highest cause of death worldwide, surpassed only by ischaemic heart disease [1]. The most devastating sub-type of stroke, intracerebral haemorrhage (ICH), accounts for 10–20% of all strokes in high-income countries, whilst incidences increase in central and East Asia and sub-Saharan Africa [2]. ICH has a mortality rate of 40% at 1-month post-ictus, coupled with a higher loss of disability adjusted life years, exceeding that of ischaemic stroke despite lower prevalence [1, 3]. Knowledge of the molecular pathophysiology surrounding haemorrhagic stroke has vastly increased in recent years, with a plethora of reviews describing the primary and secondary injury phases [4–9]. Key modifiable risk factors for ICH are well recognised, and addressing these can reduce incidence [3, 10–14]. There is still, however, a complete lack of specific treatments available to improve outcomes following ICH, despite many drugs showing efficacy in preclinical models [18]. Therefore, ICH treatment is focussed on quick diagnosis followed by reversal of anticoagulants, blood pressure management and surgery in carefully selected patients as determined by guidelines for the management of spontaneous ICH [15–18]. One reason for the current lack of specific treatments in ICH may be due, in part, to specific limitations associated with the most commonly used existing pre-clinical models of the disease, including constraints related to generating spontaneous brain haemorrhages, difficulties in observing brain pathologies in live animals and sub-optimal experimental study design [19]. It would seem, therefore, that a breakdown in the current ‘drug development pipeline’ exists for ICH. As such, there is an urgent requirement to rethink our strategies for translational ICH research so that we can investigate new therapeutic avenues for future patient treatments, as highlighted in the Haemorrhagic Stroke Academia Industry (HEADS) initiative [20].This review reinforces some of the priorities raised by HEADS, and how these may be implemented within preclinical ICH research, with our main focus highlighting the need for a more efficient, multi-directional pipeline. A more strategic approach to ensure the most appropriate models/systems are selected for each phase of the pre-clinical pipeline (such as studying the occurrence of bleed/risk factors, injury responses or treatments) would be beneficial (Fig. 1). To aid with this decision, the current pre-clinical ICH models will be discussed, including their advantages, disadvantages (see Table 1) and how translational ICH research may be potentially strengthened by adopting multiple models in parallel and embracing multidisciplinary collaboration.

**Table 1:** Comparison between different pre-clinical ICH models

Model	Haematoma expansion	Non-invasive	Spontaneity	Oedema	Intracranial pressure	Mass effect	Live imaging	Aged	High throughput	Drug testing	Behaviour assessment	Cost-effectiveness
Small mammalian												
Autologous blood	+	+	N/A	++	+++	+++	++	++	++	+++	+++	++
Collagenase	+++	+	N/A	++	+++	+++	++	++	++	+++	+++	++
Hypertension	N/D	+++	++++	++	++	++	++	++	++	+++	+++	++
CAA	N/D	++++	++++	++	++	++	++	++++	++	++	+++	+
*Col4a1*	N/A	++++	++++	++	++	++	++	++	++	++	+++	++
In vitro	N/A	N/A	N/A	N/A	N/A	N/A	++++	N/A	++++	+++	N/A	++++
Zebrafish												
Genetic	++	++++	++++	+	N/A	N/A	++++	+	++++	+++	++	++++
Chemical	++	++	++++	+	N/A	N/A	++++	+	++++	++	++	++++
Large mammalian												
Sheep	+++	+	N/A	++++	++++	++++	++	++	+	++	++	+
Pig	+++	+	N/A	++++	++++	++++	++	++	+	++	++	+

The 12 chosen factors are important when deciding which model, or combination of models can be used, to utilise the advantages of each. Whilst experimentation on sheep and pig can encompass both collagenase and autologous blood, those methods of implementing an ICH follow the same trends as observed in the rodents. *N/D* not determined, *N/A* not applicable

## In Vitro Models

In vitro studies dominate the start of the pre-clinical pipeline, where they are commonly used to complement the work from in vivo studies, especially when a pharmacological agent is being tested in rodents. The in vitro work can provide vital information on the mechanism of action, and the effects of the compound on a particular cell type, thus demonstrating the advantages of drug screening in vitro [21–28]. Typically, primary cortical neurons, microglia, astrocyte, mixed glia cultures or endothelial cell cultures are used to emulate the effects of blood on various brain specific cells that are affected following ICH. This can be beneficial for characterising the subsequent response to blood molecules in individual cell types, but less useful for identifying interactions between different brain resident cells. Further advantages of using cell-based systems include the relative ease of genetic manipulation, by using siRNA, gene editing, adenoviruses or plasmid transfections to knock down or over-express various anti- or pro-inflammatory genes or receptors, thus enabling further characterisation of the neuroinflammatory response in a relatively small time period [23, 25–27, 29].

The most conventional approach to producing an in vitro model of ICH is by stimulating the cells with a blood component to mimic the effects of intraparenchymal haemorrhage on brain resident cell types. The serine protease thrombin is activated during coagulation, and has been used to stimulate microglial cultures to determine their response to transforming growth factor β1 (TGF-β1) [30]. Oxyhaemoglobin (oxyHb) and haemoglobin (Hb) have been used to increase understanding of less common cell death mechanisms, such as necroptosis and ferroptosis [23, 24]. Furthermore, whole blood from donor mice has been used with the aid of a porous membrane insert to stimulate primary cortical neurons to allow observation of the full haemotoxic response, specifically erythrocyte lysis and haemoglobin release [31]. However, the most widely used method utilises haemin, the oxidised form of haem, which upon erythrocyte lysis is released from haemoglobin and has been shown to contribute to secondary brain injury following ICH [21, 22, 24–29, 32–34].

One challenge associated with these in vitro models is maintaining relevance to the complexity of the human condition. Following ICH, brain tissue is exposed to approximately 10 mM of haemin, whilst the concentrations used in vitro rarely exceeds 100-μM haemin [29]. Furthermore, individual cell types can exhibit differences in their sensitivity to haemin. This may potentially confound downstream analyses, but could also provide interesting insight into specific functions of particular cell types. For example, microglia are less vulnerable to haemin due to their ability to upregulate inducible nitric oxide synthase and haem oxygenase-1 [33]. Goldstein and colleagues hypothesised that in an intact central nervous system (CNS), the toxicity propagated by haemin could be dampened by endogenous antioxidants or other compounds which are not present in these specific cultures, thereby alluding to why such low concentrations of haemin has to be used experimentally [34]. Moreover, only stimulating cells with one blood component do not allow for the full effects of haemotoxicity to be observed, and the time course by which the brain resident cells become affected. Ultimately, simple cell models cannot accurately portray the haematoma expansion or many of the complex mechanisms of primary and secondary injury, hence why they are so often performed alongside in vivo rodent studies.

## Small Mammalian Models

The most widely used animals for experimental ICH research are rodents. Although rabbit models also exist, they are predominantly used for subarachnoid haemorrhage research, and as such, will not be discussed here [35–38]. A PubMed assessment of the literature using the search terms ‘intracerebral haemorrhage in rats’ and ‘intracerebral haemorrhage in mice’ identified 497 studies published since 2015. These studies could further be divided into two categories: those characterising the pathophysiology of ICH (*n* = 303); and those evaluating candidate therapies (*n* = 194). Despite a large number of the latter reporting beneficial effects, none of the treatments have as yet translated successfully to the clinic. Based on these observations, it would appear therefore that the current translational pipeline is not working. The vast majority of rodent ICH models are non-spontaneous and involve invasive techniques to generate a bleed, through stereotaxic injection of autologous blood or collagenase, and thus do not precisely mimic the human disease, as described below.

### Autologous Blood Injection Model

The autologous blood injection model was developed as a controllable and reproducible animal model of ICH, and has undergone many cycles of optimisation since its introduction in 1984. Previously, Ropper et al. had used arterial blood from a donor rat injected into the right caudate nucleus of a recipient rat in order to observe differences in regional blood flow [39]. The use of donor blood quickly became obsolete, leading to the advent of autologous blood taken from the femoral artery before injection into the caudate nucleus [40]. Currently, the most successful model involves a stereotactic injection of autologous blood at two stages into the caudate nucleus. This double injection, first implemented in 1996, was used to allow a small amount of blood to clot, in order to prevent backflow along the needle track, enabling the remaining volume of blood to emulate the haematoma [41]. Following its success in rats, the model has also been implemented for use in mice, where similar effects have been elicited [42–44].

This model is useful at recapitulating a single bleed, which is easily reproducible, and mimics many of the key traits observed in the human condition, such as mass effect, brain oedema, neurological deficits and neuroinflammation [42, 43, 45, 46]. The neuroinflammatory response includes innate immune cell infiltration, activation of brain resident immune cells, oxidative DNA damage and pro-inflammatory cytokine release, all of which have been identified as potential therapeutic targets [43]. However, the autologous blood model lacks the spontaneous nature of haemorrhage, and cannot be used to research haematoma expansion, which is present in 1/3 of patients during the first 24-h post-ictus [7]. Therefore, the primary use of the autologous blood model is to investigate the direct effects of haemotoxicity on the brain.

### Collagenase Injection

The collagenase model is the most commonly employed technique for inducing ICH in rodents. This involves the injection of collagenase, a metalloproteinase that degrades interstitial and basement membrane collagen, into the brain via stereotactic injection [47]. Collagenase causes disruption of the basal lamina of the desired cerebral arteries, resulting in blood leaking through the vessels, first achieved in rats by Rosenberg and colleagues in 1990 [47]. Bleeding was observed from 10-min post-injection, with the haematoma volume correlating to the concentration of collagenase used. Various alterations to this model can be made depending on the research question. For example, different brain locations can be injected, although the most commonly used is the right basal ganglia, which reflects the high proportion (35–70%) of patients who develop a ‘deep’ bleed [48, 49].

As with the autologous blood injection, there are caveats associated with the collagenase model, primarily revolving around the potential neuroinflammatory properties of the enzyme [50, 51]. However, in vitro studies have demonstrated that collagenase alone does not activate microglia, alter prostaglandin E2 production or induce apoptosis [51, 52]. Furthermore, in comparison to the autologous blood model, there is not an increased neuro-immune response attributed to collagenase, as both models display comparable temporal patterns of inflammation [52, 53]. Another disadvantage with the collagenase model is the difficulty in obtaining a relevant sham. Current practice uses a sham model where saline is injected into the brain [54]. However, one could argue that a better sham would be through injection of inactivated collagenase into the brain, thus controlling for potential collagenase-induced inflammation.

Comparative studies between the autologous blood and collagenase model have several key findings. The collagenase injection produces a greater primary injury, most likely attributed to the haematoma expansion which can occur in this model. Interestingly, despite autologous blood generating a more concentrated blood volume and greater initial mass effect, the collagenase model still induces more cell death, oedema and inflammation [45, 55]. There is conflicting literature regarding neurological impairment, which is suggested to be more sustained in the collagenase model, thus rendering it advantageous when observation of long-term deficits of ICH is desirable [45]. Contrastingly, another group saw more long -term motor impairments in the autologous blood model [55]. Ultimately, choice of model depends on the research question being asked. However, the translational relevance of these models has been called into question, thus reiterating the need to develop less invasive and more spontaneous techniques, or coupling these models with other experimental methods in an attempt to more closely recapitulate human ICH [20].

### Spontaneous Haemorrhage Models

Although less common, there are rodent models that haemorrhage spontaneously, thus negating the need for surgery, and potentially mimicking the clinical risk factors of ICH more closely. As such, these may be considered as more translationally relevant pre-clinical models, although the location of the bleed and haematoma size cannot be controlled and are often inconsistent between animals, as opposed to the surgically induced ICH models. The main models discussed in this section represent major risk factors for ICH in patients: hypertension, cerebral amyloid angiopathy (CAA) and cerebral small vessel disease (CSVD). The HEADS committee also highlighted the need to develop models of modifiable and non-modifiable risk factors, such as alcoholism and ageing to further understand how ICH can be prevented in these populations, as there are relatively few papers which utilise these risk factors [20, 56–63].

### Hypertension

In the 1970s, Okamoto and Aoki generated the spontaneously hypertensive stroke prone (SHRSP) rat model, associated with cerebral lesions that encompassed a wide range of microinfarcts, petechial haemorrhages and larger haemorrhages in locations such as the cerebellum [64, 65]. Furthermore, after the addition of a high salt diet or inhibitor of nitric oxide synthase Nω-nitro-L-arginine methyl ester hydrochloride (L-NAME), the SHRSP rats exhibited larger haemorrhagic events, which were coupled with ischaemia in the L-NAME-treated animals [66, 67]. Ahmad found that after L-NAME treatment, angiotensin receptor antagonist delayed the onset of stroke, thus identifying a potential pathway which may be implemented in hypertension-induced stroke [67].

Following on from the SHRSP rats, Lida and colleagues were the first group to engineer a model of spontaneous ICH in hypertensive mice, where the location of the bleeds identified were similar to those found clinically, such as the brain stem, cerebellum and basal ganglia [65]. Chronic hypertension was achieved by creating a double mutant, overexpressing the human renin and angiotensinogen genes. The mutants were also fed a high salt diet, coupled with L-NAME. A caveat with this study was the survival rates of these mice, which all died within 10 weeks, thereby preventing the ability to observe full recovery following ICH.

The same group then modelled hypertension using a non-transgenic approach by adding L-NAME to the drinking water to induce chronic baseline hypertension, followed by infusion of angiotensin II or norepinephrine to generate an acute hypertensive spike, which was successful at inducing ICH in similar clinical locations to their previous study [65, 68]. This spontaneous model has been valuable in increasing the mechanistic understanding of hypertensive ICH, with particular focus on the role of superoxide and lysyl hydroxylase 3, and how targeting these may act as an intervention to prevent ICH in hypertensive populations [69, 70]. Furthermore, RNAseq analysis from the cerebral vessels of this hypertensive mouse model identified potential key biomarkers for hypertension-induced ICH, including cancer-related pathways, mitochondrion and MHC II proteins, which may help with ICH diagnosis, although these observations need to be confirmed in hypertensive ICH patients [71].

### CAA

Rodent models of CAA differ predominantly in the mutated region of the amyloid precursor protein (APP) gene, but they all develop amyloid deposits that bind to the blood vessels and disrupt neurovascular integrity. CAA is a large risk factor for ICH patients above 70 years old; therefore, to mimic the clinical scenario, older animals are utilised in these studies. [14, 20].Winkler et al. were the first group to show that mutations in APP23 mice overexpressing APP_751_ with the Swedish double mutation (K670N/M671L) under the control of a neuron-specific-Thy-1 promoter, exhibited evidence of brain haemorrhage in 27-month old mice [72]. The haemorrhage sites correlated with the CAA vessels, providing rationale for CAA being the driving force behind vessel rupture and bleeds in these transgenic mice. Similarly, a Dutch mutation mouse (E693Q) was generated in the same way, also using APP_751_. These mice exhibited haemorrhages at 29 months, although the location and frequency of bleeds were not described [73]. Following on from this, a Swedish, Dutch and Iowa (D694N) triple-mutant AβPP_770_ mouse was produced, also developing microbleeds [74]. Whilst the frequency of these bleeds was low, this could be due to the mice being sacrificed at 12 months, and so with a longer life span, it is possible that occurrence of bleeds would have increased. Another study using the Swedish double mutation highlighted the significant increase in the number of microbleeds between 15- and 24-month old mice, further reiterating the need to prolong experimentation when using these models [75].

Some groups have explored the combination of CAA mice and hypertension, which illustrates the importance of investigating multiple risk factors for ICH together, as a patient will often present with a number of comorbidities which can work synergistically to cause a bleed. Over-expression of APP_695_ with the Swedish mutation replicated Alzheimer’s pathology, whilst L-NAME treatment and a brief angiotensin II infusion created chronic hypertension. Subsequently, transient acute hypertension was generated by daily doses of angiotensin II. The resulting hypertensive CAA mice had an increased susceptibility to spontaneous ICH at 15 months old [76]. Whilst this is very successful at producing an all-encompassing spontaneous model, major caveats are associated with increased technical demand of generating sufficient hypertension, coupled with the prolonged study time.

### Col4a1

Collagen type IV is an integral part of the basement membrane, and is pivotal in providing structural support to tissues. Mutations in the isoform *COL4A1* have been identified in CSVD, haemorrhagic stroke (particularly in younger individuals), familial porencephaly and aneurysm formation of the carotid artery [77]. Newborn mice with mutations in *Col4a1/a2* also exhibit haemorrhage phenotypes. Similarly, older mice experience structural defects in large calibre arteries, as observed in the descending aortae, where focal separation of the endothelium from the media altered the vascular smooth muscle and endothelial cell function. It is hypothesised this may also occur in other large calibre arteries such as the carotid, and may provide rationale for the increased likelihood of ICH in these animals [77]. Another group recreated different *Cola4a1/a2* mutations in mice to understand the genetics and mechanisms that can lead to bleeding, in the hope to improve patient prognosis and treatment for the specific mutations [78]. They also identified several modifiable risk factors which may increase the risk of developing an ICH in this population, such as anticoagulant treatment, acute exercise and vaginal delivery at birth, although these factors would need to be considered on an individual basis, rather than a blanket statement for all *COL4A1* patients [78]. Recently, a *Col4a1* mutant mouse model has been used to mimic deep spontaneous haemorrhages which can occur in patients [79]. A novel segment (transitional segment) was identified between arterioles and capillaries which was hypermuscularised in the mutant mice, thought to play a role in the development of ICH due to subsequent increased intravascular pressure in the upstream feeding arteriole. This study also utilised post-mortem (PM) brain tissue from deep ICH fatalities to corroborate the mouse data. Therefore, this study is a prime example of how using different systems (i.e., mouse models + PM material) can increase confidence in the translational relevance of pre-clinical discoveries.

Despite poor clinical translation in terms of new treatments, the usefulness of both surgical and spontaneous ICH models cannot be disputed, as they have proven vital in studying the multi-faceted effects of ICH within a whole organism. Rodent ICH models have provided the ability to investigate the effects of neurotoxic insults from blood, the infiltrating immune response, neurological deficits and behavioural alterations. However, with the autologous blood and collagenase model, the surgical procedures required to do this are highly invasive and have limitations on how well they truly mimic the clinical scenario. Indeed, the side effects relating to the stress associated with anaesthesia and surgery will undoubtedly confound some aspects of downstream outcome analysis. Moreover, in the past, a disadvantage of rodent models was the presence of the mammalian skull preventing observation of the pathophysiology in real time. However, now various imaging techniques can be implemented such as MRI, PET and laser-speckle. Whilst MRI is commonly used following ICH-induced surgery to view haematoma expansion and/or resolution, and laser speckle to characterise the dynamic changes in blood flow, they are not utilised in identifying bleeds in spontaneous models [55, 80–84]. This is especially apparent in the long-term studies associated with CAA, where the brain is not observed until the animal is 2–3 years old. Prussian blue staining of hemosiderin in the brain is the sole marker used for identifying cerebral bleeds, so the timing of the vessel rupture is largely unknown [72–75]. Furthermore, spontaneous models are rarely used for drug testing, which would be useful to find preventative treatments, or to alleviate symptoms following a bleed, especially as a large proportion of ICH patients suffer from the risk factors modelled in these animals.

## Zebrafish Models

Some of the limitations associated with the in vitro and rodent models can be compensated for by the emerging use of zebrafish (*Danio rerio*) models, which can be thought of as an intermediate between in vitro and higher order in vivo systems. Zebrafish possess many benefits as an in vivo model, such as rapid development, transparency of embryos and larvae, non-invasive in vivo imaging and ease of genetic manipulation [85]. Furthermore, as a vertebrate species, the zebrafish genome shares ~ 70% homology with humans thus making them an advantageous system to complement mammalian models [86]. We recognise that zebrafish larvae are developing animals, and different disease mechanisms may exist in comparison to adult humans. However, we have shown that key characteristics associated with the pathological response to blood in the brain are apparently conserved between young fish and adult humans, suggesting they can be reliably used to model aspects of ICH [87, 88].

In terms of ICH, one of the primary benefits of using zebrafish larvae is that the haemorrhage is produced in a non-invasive manner, thus arguably mimicking the spontaneous nature of human ICH more closely than the surgical rodent models. Several different genetically modified lines exist where haemorrhages are established in this way, whilst chemical induction is another common approach for inducing a brain bleed. There are 2 primary mechanisms by which a bleed can be induced: blood brain barrier (BBB) defects and weakness in the developing blood vessels. With the former, mutations in *notch3*, a regulator in brain pericyte proliferation can result in impaired BBB function and increased frequency of brain haemorrhages [89]. As such, this model may also be useful for studying aspects of CSVD.

Nascent cranial vessel weakness is induced by targeting components important in the stabilisation and development of the cerebrovasculature. βPix is a protein encoded for by the gene *arhgef7b*, thought to play a role in vascular stabilisation. Mutation of βPix results in ICH and hydrocephalus in zebrafish larvae, thus generating the nickname ‘bubblehead’ for these fish/alleles [90]. Similarly, mutation of the Pak2a protein results in a different, though comparable zebrafish model of ICH, known as the ‘redhead’ mutant [91]. Defects in primary cilium attached to endothelial cells have been shown to disrupt cerebral-vascular integrity, resulting in ICH in the intraflagellar transport mutant [92].

Aside from genetic alterations to evoke weakness in the neurovasculature, pharmacological agents can be utilised to induce a haemorrhage in zebrafish larvae via water bath incubations and absorption. Statins are most commonly used for this and act by inhibiting the cholesterol biosynthesis rate limiting enzyme 3-hydroxy-3-methylglutaryl-coA reductase (HMGCR). This leads to altered signalling via geranylgeranyl pyrophosphate (GGPP) and reduces activation of Rho GTPases, which act to regulate vascular permeability [93, 94]. Importantly, transient gene knockdown of *hmgcra* using a morpholino (MO) phenocopies, the haemorrhages were observed with statin treatment [94]. Targeting HMGCR not only provides a quick and reliable method of inducing ICH in zebrafish larvae, but may also afford insight into the clinical association between hypocholesterolaemia and increased ICH risk [95–99]. However, statin treatment has been shown to alter myogenesis in developing zebrafish larvae alongside reducing locomotion and heartbeat, which could potentially confound other mechanistic studies which aim to focus solely on the effects of ICH on the larvae [100].

Furthermore, there are rare genetic conditions associated with ICH which cannot be accurately represented in rodents, but can be in zebrafish. An example of this is the rare autosomal recessive interferonopathy: Aicardi-Goutières syndrome subtype 5 (AGS5). Mutations in the viral restriction factor protein SAM and HD domain containing Deoxynucleoside Triphosphate Triphosphohydrolase 1 (SAMHD1) produce an exaggerated type I interferon (IFN) response and cerebrovascular disease in some patients [101–105]. Rodent models of AGS5 exist, but these mice lack any overt physical phenotype [106, 107]. Contrastingly, a MO knockdown of the *samhd1* gene in zebrafish larvae resulted in spontaneous ICH and a significant upregulation of type I IFN [108]. Similar to AGS, another genetic autoimmune disease has been characterised in zebrafish: deficiency of Adenosine Deaminase 2 (ADA2). ADA2 encompasses systemic inflammation and a vascular phenotype arising in childhood, which can result in ischaemic or haemorrhagic cerebral events. A mouse orthologue of the gene encoding ADA2 (*CECR1)* does not exist; however, zebrafish express two paralogues of the *CECR1* gene: *cecr1a* and *cecr1b.* Following MO knockdown of *cecr1b*, these morphants exhibited intracranial bleeding, thus alluding to a role of ADA2 in cranial vessel development or integrity [109].

The use of zebrafish as a disease modelling tool is increasing, and as previously mentioned, they possess several key benefits for studying ICH. However, this is not to suggest that zebrafish should replace any of the well-established models, rather we propose that they should be utilised alongside other in vitro and in vivo studies. For example, using zebrafish for large-scale drug screens, and taking forward the positive hits to interrogate further in mammalian models may increase efficiency of drug development. Furthermore, this approach may also have ethical implications by reducing the numbers of mammals used for primary drug screens. Zebrafish represent a powerful model system for large drug screening studies because of their relatively high-throughput nature, and the high conservation of drug binding sites [110]. There are, however, some caveats associated with zebrafish larval models, such as the lack of skull preventing changes in intracranial pressure and mass effect to be observed following ICH. In addition, as recovery rates are so rapid in zebrafish larvae as demonstrated by Crilly et al., it poses questions on how this may differ to recovery in adult humans. However, understanding these types of processes following ICH during development may provide clues into how we might consider recovering the aged human brain in the future [87].

## Larger Mammalian Models

One recurring issue with the animal models described above is their size in comparison to humans, and thus it is difficult to assure accurate translation because of obvious structural differences between rodents, zebrafish and human brains. One approach to address this is through the use of larger animal species, with brains more comparable to humans, in terms of size, white:grey matter ratios and the presence of gyri. The only 2 species discussed in this section are ovine and swine models; however, this is not to purposefully ignore any additional large mammalian models of ICH. The use of monkeys, canines and cats to study ICH has become largely obsolete, due mainly to ethical considerations [111, 112]. As the main premise of this review is to encourage collaborations between different ICH disciplines, it was thought best to include only those species which are widely used and more broadly accessible.

Boltze and colleagues [113] recently replicated the autologous blood model in adult sheep, observing similar histopathological observations as found in rodent models, such as brain resident cell recruitment and axonal damage. Whilst this preliminary study was used to demonstrate how ovine models can be used as a successful pre-clinical model of ICH, in the future, it is hoped that they will provide additional translational benefits when studying surgical interventions, for example like those performed in the MISTIE and STITCH trials [113–118]. Such candidate intervention techniques are infrequently implemented in other pre-clinical models, and due to the small size of brain and skull, it may be more desirable to perform these types of surgical intervention techniques in an animal model with a larger head [37, 119, 120]. Furthermore, as an aside, this was one of the few pre-clinical ICH studies to include both male and female animals, which as determined by the HEADS initiative needs to be performed more frequently across species, as it is unethical to automatically exclude half of the world’s population because female experimental outcomes may differ from males [20]. The other existing pre-clinical studies experimenting on female animals have been used to mainly view hormonal differences between sexes and also how various treatment alters outcome following ICH [82, 121–125].

Swine models of both autologous blood and collagenase injection are implemented for many of the same reasons as ovine models, most notably when characterising the primary injury phase, as comparable haematoma volumes to humans can be produced, making it more beneficial to study mass effect and the mechanism of oedema formation in these larger animals [126–128]. In addition, drug treatments can also be tested in swine models, such as the iron chelator deferoxamine, where it was shown to reduce perihaematomal iron accumulation, neuronal cell death and white matter injury, comparable to its effects in a rat model of ICH [129]. However, the recent i-DEF clinical trial on deferoxamine gave rise to neutral results, thus we should utilise these larger mammalian models to optimise treatment protocols before clinical trials, such as determining the most effective way to administer a drug [130].

Surgical intervention has already been tested in a swine model, whereby following an autologous blood injection, tissue plasminogen activator (tPA) was added to lyse the clot, and the haematoma was aspirated out of the brain, resulting in a significant reduction in oedema [131]. Aside from primary injury, functional changes which occur during ICH can also be measured in the swine model, including alterations within the primary somatic evoked potentials and the resulting cortical spreading depression, which were found to be similar to what was found in a rat model previously [132].

Use of farm animals in ICH research is extremely important to understand the pathophysiology that occurs in a larger brain. However, one limitation of these models in comparison to rodents relates to a relative lack of behavioural assays currently available for measuring neurological outcomes. Furthermore, due to most pre-clinical research being directed towards rodents, it means there is a scarcity of species-specific reagents available for other models, such as sheep, pig and zebrafish, perhaps hindering the ability to look at the secondary injury response as thoroughly as can be observed in mice and rats [128]. The experimentation time is often longer than that of rodent work due to the use of older animals, leading to increased costs, and imaging capabilities are essential. The latter requires infrastructure and resources that few centres currently have.

## Post-Mortem/Ex Vivo Studies

Ex vivo studies using PM brain tissue from patients who have died from ICH are surprisingly infrequent, but represent a precious and most clinically relevant source of material. Fortunately, in some instances, what is observed in human samples is comparable to findings in pre-clinical models, as seen in a small proportion of studies [79, 133, 134]. Aside from comparative studies, Wu and colleagues looked at the expression levels of nuclear factor-kappa B (NF-κB), macrophage inflammatory protein-2 (MIP-2) and matrix metalloproteinase-9 (MMP9) using immunohistochemistry. This was successful in enhancing understanding of the time course of brain inflammation following ICH in PM tissues [135]. Furthermore, the robust nature of PM tissue means it can be used for purposes other than just viewing the morphological and cellular characterisation of ICH brains, as demonstrated by a group who performed RNAseq analysis on the frontal and occipital lobes to understand gene expression in hereditary cerebral haemorrhage with amyloidosis-Dutch type (HCHWA-D) [136].

Whilst researching the effect of stroke on the brain is paramount, studies which utilise stroke patient blood can be extremely useful for immune profiling and ‘omics-based analyses. This was recently performed by Stamova and colleagues who used RNA from both ischaemic and haemorrhagic stroke patients to reinforce the molecular differences between each condition [137]. Moreover, examining serum from ICH patients has identified the dysregulation of various pro and anti-inflammatory proteins, which have also been confirmed in rodent ICH models [70, 138, 139]. Additionally, Taylor et al. used ICH patient plasma to determine the role of TGF-β1 following haemorrhage. This ex vivo approach was coupled with both in vitro and in vivo experiments, demonstrating the power of using multiple models/systems, to corroborate discoveries and increase confidence in their validity [30]. Similarly, Lu et al. have recently utilised these three pre-clinical model systems when investigating the effects of the microRNA miR-181c following ICH [140].

There are, however, some problems with PM brain tissue, dependent on factors outside of the researchers’ control, such as the availability of particular brain regions, and also the time that the brain is fixed following death [135, 141]. The latter was shown to cause potential exaggeration of glial cell swelling, which is thought to be attributed to the fixation process in devitalised tissue, also seen in delayed fixation in rat brain [141]. Ultimately, the number of studies using PM and ex vivo tissue is minimal. Future work should focus on incorporating these valuable resources into studies, which may become more accessible if cross-discipline collaborations develop, for example, between basic scientists and brain banks.

## Conclusion

**Fig. 2:**
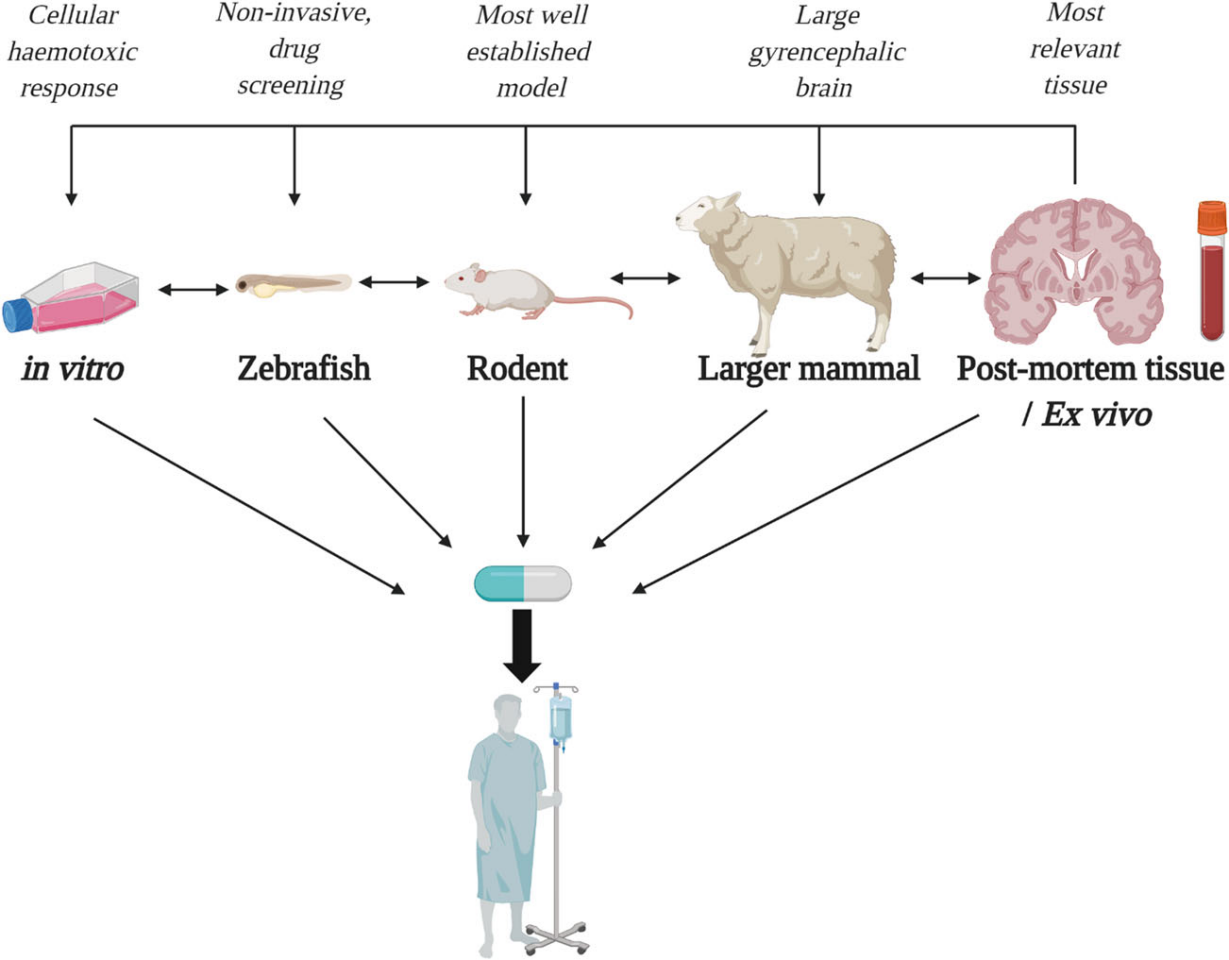
Multidirectional pipeline of preclinical ICH research. In this pipeline, there is both a feedforward and a feedback loop, to ensure any new findings are valid and observed across multiple models. In addition, the use of post-mortem and ex vivo tissue should feedback into all other pre-clinical models in an attempt to maintain clinical relevance. Fostering multi-discipline collaborations increases the likelihood of creating a drug which will be successful in humans, especially if it is shown to exert positive effects across the pipeline. Maximal advantages are denoted above each model/system

In this review, we have discussed the multiple models of ICH that exist to inform about a highly disabling condition, currently with no specific medical treatment. To facilitate successful translation in the future, it is clear that there needs to be greater collaboration within the pre-clinical ICH research community to enable cross-talk between studies using different models and by utilising the advantages of each system. The most common multi-model papers exhibit experimentation on rodent and in vitro systems. Moving forward, we suggest a bi-directional 2- or 3-pronged approach for pre-clinical ICH research. For example, using the autologous blood injection rodent model, the thrombin in vitro model, and also incorporating an ex vivo element to the study, as so elegantly illustrated by both Taylor, Lu and colleagues recently [30, 140]. These studies illustrate the power of using multiple systems to investigate a particular question, an approach that will hopefully become more frequently adopted for translational ICH research. Moreover, as one of the key criteria identified by the HEADS committee, we also believe it is essential for pre-clinical findings to be tested and verified in at least two different species (and not both rodents) [20]. By replicating the effect of a drug or compound identified in a smaller animal model in a higher order species such as sheep or pig, it increases the confidence that such a treatment may be efficacious in humans and worthy of consideration for clinical investigation. Furthermore, it is also apparent that the use of brain banks for PM tissue has not been utilised enough for ICH research. Human PM tissue is ideal to validate data obtained from animal models whilst also advancing our understanding of the human pathology and how it may be targeted. Therefore, the use of PM tissue should be implemented when possible, and can work in conjunction with any of the other models (see Fig. 2)*.* Similarly, serum from ICH patients is also understudied, again reinforcing the need for increased cross-talk between pre-clinical scientists and clinicians to obtain this type of material, and to understand key differences which may be observed between comorbid populations which go on to develop an ICH and those that do not. This work would feed into the other pre-clinical work taking place, and can provide validity for the other models, if similar trends are observed. By fostering collaborations with different disciplines, it will strengthen the depth and breadth of ICH research, and will increase the chances of finding new treatment options for ICH patients in the future.
